# A Versatile and Ultrasensitive Electrochemical Sensing Platform for Detection of Chlorpromazine Based on Nitrogen-Doped Carbon Dots/Cuprous Oxide Composite

**DOI:** 10.3390/nano10081513

**Published:** 2020-08-01

**Authors:** Venkata Narayana Palakollu, Rajshekhar Karpoormath, Lei Wang, Jiao-Ning Tang, Chen Liu

**Affiliations:** 1 Shenzhen Key Laboratory of Polymer Science and Technology, Guangdong Research Center for Interfacial Engineering of Functional Materials, College of Materials Science and Engineering, Shenzhen University, Shenzhen 518055, China; palakollu@szu.edu.cn (V.N.P.); wl@szu.edu.cn (L.W.); tjn@szu.edu.cn (J.-N.T.); 2 Key Laboratory of Optoelectronic Devices and Systems of Ministry of Education and Guangdong Province, College of Optoelectronic Engineering, Shenzhen University, 3688 Nanhai Ave, Shenzhen 518060, China; 3 Department of Pharmaceutical Chemistry, College of Health Sciences, University of KwaZulu-Natal, Westville Campus, Durban 4000, South Africa; karpoormath@ukzn.ac.za

**Keywords:** electrochemical sensor, carbon dots, cuprous oxide, chlorpromazine, antipsychotic drug, pharmaceutical formulations

## Abstract

The excessive intake of chlorpromazine (CPZ) adversely affects human health profoundly, leading to a series of severe diseases such as hepatomegaly and dyskinesia. The rapid and precise detection of CPZ in real samples is of great significance for its effective surveillance. Herein, a versatile and sensitive electrochemical sensor was developed for the detection of antipsychotic drug CPZ based on a Nafion (Nf)-supported nitrogen-doped carbon dots/cuprous oxide (N-CDs/Cu_2_O) composite. The as-synthesized N-CDs/Cu_2_O composite was systematically characterized using various physicochemical techniques. The developed composite-based sensor displayed excellent performance towards CPZ determination in a dynamic linear range of 0.001–230 µM with the detection limit of 25 nM. Remarkably, the developed sensor displayed good performance in terms of sensitivity and selectivity. Furthermore, good anti-interference properties toward CPZ determination were attained despite the presence of highly concentrated interfering compounds. Therefore, this composite could be a notable potential modifier to enhance electrocatalytic activity onto the surface of the electrode. Finally, N-CDs/Cu_2_O/Nf-based sensor was effectively applied for quantification of CPZ in human urine and pharmaceutical formulation samples.

## 1. Introduction

As a psychosis medication and the first-generation antipsychotic drug used for the management of schizophrenia, chlorpromazine (CPZ) is used to control various mental disorders, hypoxia, psychomotor agitation, manic depression, paranoia, anxiety, and tension [1]. However, it is frequently used as a growth promotion agent for the livestock industry. The illegal overuse and wrongful administration of CPZ results in its chronic accumulation in the environment and human food, which could lead to severe health issues such as hematological disorders, central nervous system reactions, and hypotensive effects [2,3,4]. Therefore, a highly sensitive and selective tool for the emerging, rapid, and accurate sensing of CPZ is of great significance to the field of pharmacological research.

Over the last few years, various techniques have successfully been reported for determining the content of CPZ in pharmaceutical formulations such as chemiluminescence [5,6], chromatography [3,7,8], electrochemiluminescence [9] and flow injection [10]. However, compared with the electrochemical technique, which offers good sensitivity and selectivity, rapid response, portability and low cost [11,12,13,14], the aforesaid methods have non-negligible demerits such as the requirement of sophisticated instruments, complicated routine analysis, and specially trained technicians [15,16,17]. 

Cuprous oxide (Cu_2_O) is known as one of the desired and ideal materials in the electrochemical sensing applications not only due to the natural abundance and low cost but also its higher catalytic activity, good chemical stability, environmental compatibility and non-toxicity [18,19]. Owing to the limited sensitivity and narrow-range liner detection of pristine semiconductor Cu_2_O sensors, integrating carbonaceous materials has been regarded as one of the efficacious ways that can compensate the defects and drastically enhance the electrical conductivity. Carbon dots (CDs) are a type of carbon nanoparticles with sizes less than 10 nm and have recently attracted great attention because of their high surface area ratio, excellent chemical and thermal stability and good electronic conductivity [20,21]. This carbonaceous material is progressively being employed to enable the electron-transfer at the interface of electrode surface and analyte in the electrochemical processes, which results from the plenty of hydrophobic planes and hydrophilic edges in the CDs that can escalate the interaction of analyte and electrode [22]. Moreover, the modification of carbon core, functionality and doping hetero atom could further influence the electrocatalytic activity of CDs [23]. In particular, doping nitrogen in the CDs (N-CDs) is an effective way to significantly improve its electrical conductivity and increase the binding active sites as well as ameliorate the electron-donor performance [24,25], endowing N-CDs excellent performance in the area of biomedical applications such as bioimaging and biosensing [26]. Jahanbakhshi and Habibi [27] developed an electrochemical sensor for H_2_O_2_ based on gold nanoparticles/CDs nanohybrid. The investigation revealed that the nanohybrid possesses good electrochemical performance towards the reduction of H_2_O_2_. Recently, many Cu_2_O-based materials have been employed for electrochemical sensing and biosensing applications due to the properties such as high catalytic activity and strong electroactive surface area [25,28,29]. Assimilating unique properties of each material and their features would help in improving the performance of sensor applications. Hence, it would be exciting to bring out novel properties by taking the benefits of both Cu_2_O and N-CDs.

In this work, we developed in situ hybridized Cu_2_O with N-CDs (N-CDs/Cu_2_O) composite via a simple two-step process through hydrothermal and wet chemical approaches. The developed composite was used as sensing materials with the support of Nafion (Nf) for electrochemical determination of CPZ, exhibiting a good electrocatalytic response towards the sensing of CPZ. More distinctively, the developed N-CDs/Cu_2_O/Nf composite modified electrode shows good selectivity, high sensitivity, and wide linear range with lowest detection limit towards CPZ determination. The proposed sensor was also employed for testing of the applicability to real samples such as quantification of CPZ in the pharmaceutical formulation as well as in urine sample without needing any additional chemical pretreatment.

## 2. Materials and Methods 

### 2.1. Materials and Reagents

Chlorpromazine hydrochloride, potassium chloride, ethylenediamine, catechol, ethanol, potassium ferricyanide, Nafion, magnesium sulfate, dopamine hydrochloride, sodium hydroxide, glucose, sodium chloride, uric acid, ascorbic acid and *N*,*N*-dimethylformamide, purchased from Shanghai Aladdin Biochemical Technology Co., Ltd (Shanghai, China), potassium ferrocyanide, copper (II) sulfate pentahydrate procured from Shanghai Macklin Biochemical Co., Ltd., (Shanghai, China), citric acid obtained from Tianjin Damao Chemical reagents (Tianjin China). Sodium dihydrogen orthophosphate and sodium phosphate dibasic were used to prepare buffer solutions. Deionized (DI) water was used throughout this work. 

The electrochemical data were recorded using CHI 660E electrochemical workstation (CH Instruments, Shanghai, China). A conventional three electrodes (a glassy carbon electrode (GCE) or a modified GCE (working electrode), a platinum wire (axillary electrode) and an Ag|AgCl (3.0 M KCl) electrode (reference electrode)) electrochemical system was used. All the experiments were performed at room temperature only. The pH measurement of buffer solutions was carried out using pH meter (Shanghai Yueping Scientific Instrument Ltd., Shanghai, China). The diffraction patterns of as-synthesized materials were collected using X-ray diffractometer (XRD (Bruker^®^ AXS D8 adv, Bruker (Beijing) Scientific Technology Co., Ltd., Beijing, China)) and the chemical compositions were attained using Thermo-Fisher^®^ Microlab 350 X-ray photoelectron spectroscopy (XPS) system (Hillsboro, OR, USA). The surface morphological studies of as-synthesized materials were studied using Hitachi^®^ Field emission-scanning electron microscopy (FESEM, SU-70, Tokyo, Japan)) with energy-dispersive X-ray spectroscopy (EDS). The Fourier transform infrared spectrometer (Nicolet 6700) was employed to study FT-IR spectra. Transmission electron microscopic (TEM) analysis was carried out using field emission electron microscope (JEOL JEM-2100F, Tokyo, Japan).

### 2.2. Synthesis of N-CDs

In a typical synthesis of N-CDs, 1.0508 g of citric acid and 335 µL of ethylenediamine were dissolved in 10 mL of DI water and then transferred to 50 mL Teflon lined autoclave and heated at 200 °C for 5 h. After the autoclave was cooled to room temperature naturally, the obtained products were centrifuged at 3500 rpm to separate unreacted and large size particles. The outcome was freeze-dried for 72 h to get solid N-CDs.

### 2.3. Synthesis of N-CDs/Cu_2_O Composite

**Scheme 1:**
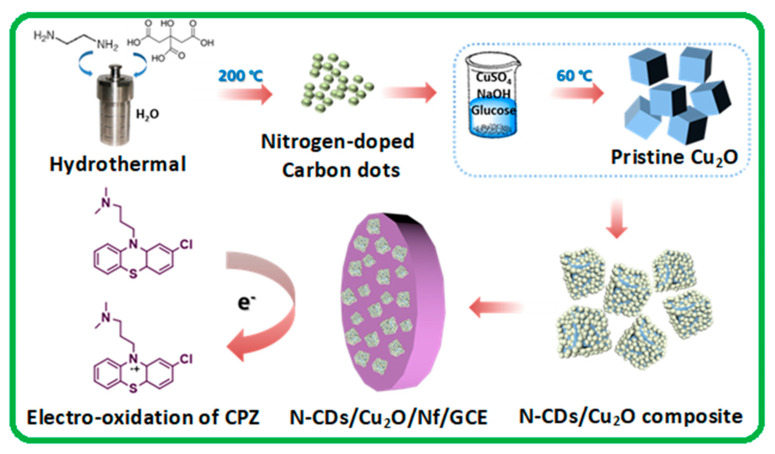
Schematic illustration of electrochemical sensor development and catalytic response of CPZ.

N-CDs/Cu_2_O composite was synthesized according to previous literature with minor modifications [30]. Typically, 500 mg of copper (II) sulfate pentahydrate (CuSO_4_·5H_2_O) and 10 mL of DI water containing 1mg of N-CDs (solid) were dissolved in 40 mL of DI water under mechanical stirring. 10 mL of 1 M NaOH was slowly added to the above solution and followed by injecting 10 mL of 0.3 M glucose (acts as a reducing agent) after being heated to 60 °C. After aging at 60 °C for 3.5 h, the product was centrifuged and then washed 3 times with ethanol and DI water, respectively. Later, the product was dried at 65 °C and termed as N-CDs/Cu_2_O (Scheme 1). The similar procedure was used to synthesize pure Cu_2_O cubes without introducing N-CDs during the synthesis. 

### 2.4. Preparation of N-CDs/Cu_2_O/Nf Composite Modified Electrode

First, 5 mg of as-synthesized N-CDs/Cu_2_O was dispersed in 5 mL of N-dimethylformamide by ultra-sonication for 20 min (solution A). Then, 20 µL of 5% Nf solution and 180 µL of ethanol were ultra-sonicated for 30 min (solution B). Next, an optimized mixture of solution A and B (1:32, v/v) were ultra-sonicated for 5 min to obtain a suspension. Afterwards, 5 µL of the suspension was dropped onto the surface of cleaned GCE and dried under the IR lamp. The resulted electrode was known as N-CDs/Cu_2_O/Nf/GCE. Similarly, 5 mg of N-CDs and 5 mg of Cu_2_O were used for preparing N-CDs/GCE and Cu_2_O/GCE, respectively.

### 2.5. Real Sample Preparation

The urine sample was collected from healthy personnel and centrifuged for 5 min at 5000 rpm to obtain supernatant solution. The obtained supernatant solution was diluted 10 times with 0.1 M phosphate buffer solution (PBS) (pH 7.0) and stored in refrigerator at 4 °C for study analytical application of proposed sensor in biological matrix.

## 3. Results and Discussion

### 3.1. Physicochemical Characterization 

**Figure 1:**
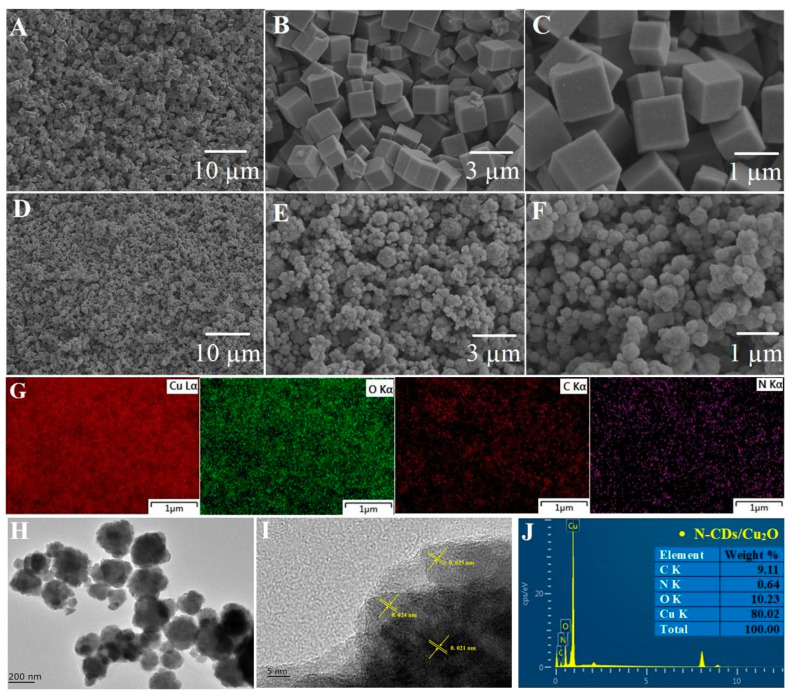
(**A**–**C**) SEM images of Cu_2_O, (**D**–**F**) N-CDs/Cu_2_O composite, (**G**) elemental mapping of N-CDs/Cu_2_O composite, (**H**) TEM, (**I**) its corresponding high resolution transmission electron microscopic (HRTEM) image of N-CDs/Cu_2_O and (**J**) elemental composition of N-CDs/Cu_2_O composite.

The morphological and structural features of as-synthesized Cu_2_O and N-CDs/Cu_2_O composite were studied using FESEM and TEM as shown in Figure 1. The pure Cu_2_O displayed a cube-like structure with an evidently smooth surface (Figure 1A–C). With the introduction of N-CDs, N-CDs/Cu_2_O composite exhibited a sphere-like morphology in Figure 1D–F due to the coverage of CDs outside cubic Cu_2_O. The elemental mapping of N-CDs/Cu_2_O composite in Figure 1G was conducted to determine the homogeneous distribution of C, N, Cu, and O elements. The Cu and O signals were much higher than that of C and N elements, indicating the cuprous oxide is dominant in the composite materials. The structure of N-CDs/Cu_2_O composite can be obviously observed in the TEM image of Figure 1H, showing as black spots of N-CDs spread on the surface of the Cu_2_O. From HRTEM analysis (Figure 1I), the lattice spacings of 0.25 nm, 0.24 nm and 0.21 nm were consistent with (111) plane of the crystallographic Cu_2_O and (200) and (100) planes of CDs, respectively, which is in well agreement with the XRD results [24,25]. The specific content of each element was further confirmed by EDS analysis in a random region of N-CDs/Cu_2_O composite, which demonstrated the element composition of 9.11% C, 0.64% N, 10.23% O and 80.02% Cu in the as-synthesized N-CDs/Cu_2_O composite (Figure 1J).

**Figure 2:**
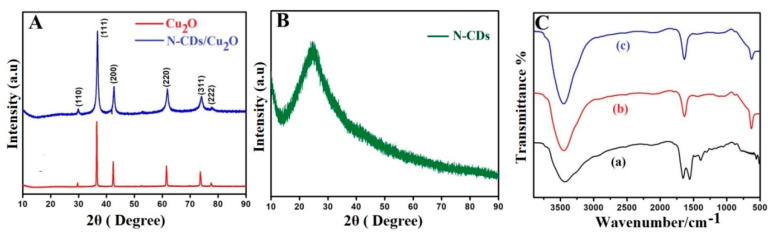
(**A**) XRD pattern of Cu_2_O and N-CDs/Cu_2_O composite, (**B**) XRD pattern of N-CDs; (**C**) FT-IR spectra of (a) N-CDs, (b) Cu_2_O and (c) N-CDs/Cu_2_O composite.

The as-synthesized Cu_2_O and N-CDs/Cu_2_O composite were characterized by XRD test and the similar diffraction peaks at 29.79°, 36.54°, 42.43°, 61.40°, 73.62° and 77.62° in Figure 2A assigned to (110), (111), (200), (220), (311) and (222) lattice planes of cubic phase Cu_2_O (JCPDS 05–0667) [31]. The existence of carbon source for N-CDs was revealed by a broad peak at around 2θ = 24.73° (Figure 2B). However, no characteristic peak of carbon source from 23° to 27° was attained for N-CDs/Cu_2_O composite due to the presence of minor quantities, well dispersions and low crystallinity of N-CDs in N-CDs/Cu_2_O composite [30]. Moreover, no additional peaks were observed in the XRD pattern of both pure Cu_2_O and N-CDs/Cu_2_O composite and confirming the high purity of synthesized materials. The functionalities of N-CDs, Cu_2_O and N-CDs/Cu_2_O composite were further investigated by FT-IR spectra. As seen in Figure 2C, the broad peaks at 3430 cm^−1^ and 3255 cm^−1^ represent O-H and N-H stretching vibrations, respectively. Moreover, bands of 1661 cm^−1^ (amide I) and 1554 cm^−1^ (amide II) are ascribed to the bending vibrations of the amide group [32]. The weak merged peaks at 2943 and 2879 cm^−1^ are related to the C–H bond stretching vibrations. All of them demonstrate the presence of N-CDs in the composite. A sharp and intense peak at 627 cm^-1^ in FT-IR spectra of N-CDs/Cu_2_O and Cu_2_O is primarily assigned to the characteristic stretching of Cu-O, which specifies the framework of Cu_2_O. Furthermore, the peak positions of N-CDs/Cu_2_O in FT-IR are almost similar to Cu_2_O, owing to small quantities of N-CDs in N-CDs/Cu_2_O composite.

The chemical composition of N-CDs/Cu_2_O was studied by XPS analysis. The XPS survey spectrum (Figure S1A) confirms the existence of elements such as Cu, N, C and O in N-CDs/Cu_2_O. The deconvoluted spectrum of Cu 2p of N-CDs/Cu_2_O composite displayed two peaks at 935.26 eV and 955.15 eV (Figure S1B), corresponding to its 2p_3/2_ and 2p_1/2_ spin-orbital, which is ascribed to Cu^+^ of Cu_2_O [33]. The difference of less than 20 eV in binding energies (Cu 2p_3/2_ and Cu 2p_1/2_) confirming the existence of Cu_2_O in the prepared composite. The deconvoluted spectrum of C 1s of composite displayed the Gaussian peaks at 284.96, 285.7, 287.5 and 290 eV (Figure S1C) are corresponding to C–C (sp^3^), C–N (sp^3^), C=O (sp^2^) and O–C=O (sp^2^) or C=N, respectively [34]. The presence of C-N bonding reveals nitrogen-doping in the CDs. The fitted two Gaussian peaks of O 1s at 531.72 and 534.0 eV (Figure S1D) are ascribed to C=O and C–OH / C–O–C groups, respectively [35]. The peaks of N 1s at 399.5, 402.79 and 405.8 eV shown in Figure S1E specify the existence of nitrogen typically in the form of pyrrolic or pyridonic-N, graphitic-N and N-oxides [36]. Pyrrolic or pyridonic-N, graphitic-N, and N-oxides can generate different electronic environments for carbon atoms and further create electrochemically active sites. Therefore, based on deconvolution results of XPS analysis, it can be evidently confirmed that N-doping was perfectly taken place in CDs. Interestingly, the presence of pyridinic-N is additionally beneficial for enhancing the electrochemical sensing of organic compounds [37]. Moreover, graphitic-N can considerably increase the conductivity of N-CDs/Cu_2_O composite through additional electrons at nitrogen sites [38].

### 3.2. Electrochemical Characterization of N-CDs/Cu_2_O/Nf/GCE 

**Figure 3:**
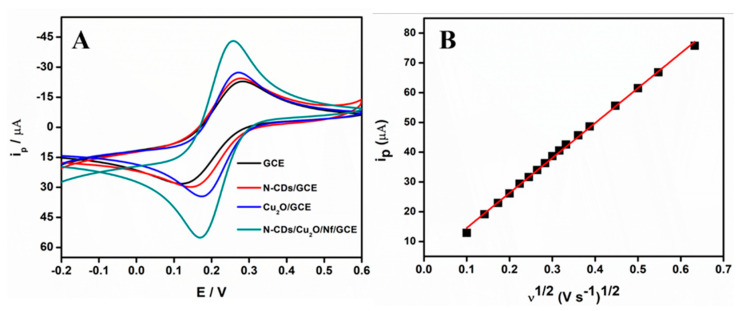
(**A**) Cyclic voltammetric responses of different modified electrodes in 0.1 M KCl containing 2.5 mM [Fe(CN)_6_]^3−/4−^ at a scan rate of 100 mV s^−1^, (**B**) Calibration plot of square root of scan rate vs. anodic peak current of 1 mM K_3_[Fe(CN)_6_] in 0.1 M KCl at N-CDs/Cu_2_O/Nf/GCE.

The electrochemical response of bare and modified electrodes was studied using CV technique, in 0.1 M KCl containing 2.5 mM of [Fe(CN)_6_]^3−/4−^ (equimolar) at a scan rate of 100mV s^−1^. Figure 3A displays cyclic voltammetric response of GCE, N-CDs/GCE, Cu_2_O/GCE and N-CDs/Cu_2_O/Nf/GCE. As can be seen, typical redox peaks were attained with peak to peak separation (ΔE_p_) of 164 mV at bare GCE due to relatively poor reversibility of GCE in [Fe(CN)_6_]^3−/4−^. The N-CDs modified GCE showed a better performance with ΔE_p_ of 134 mV with improved redox peak currents than GCE by the good electrical conductivity of N-CDs. Furthermore, Cu_2_O/GCE exhibited the ΔE_p_ of 98 mV due to the good catalytic activity of Cu_2_O. A significant increase in the peak currents with decreased ΔE_p_ (89 mV) was observed for N-CDs/Cu_2_O/Nf/GCE. Moreover, the intensities of peak currents at N-CDs/Cu_2_O/Nf/GCE were found to be 4.68 times higher than that of bare GCE. Obviously, remarkable enhancement in the peak currents, least ΔE_p_ and perfect reversibility of redox system observed in cyclic voltammogram at this composite modified electrode signifying the excellent electrocatalytic enhancement. Moreover, the anodic peak current(i_pa_)/cathodic peak current(i_pc_) was almost unity and can be treated as a significant factor to testify the stable electrochemical response for this redox system at the modified electrode. This has probably resulted from the synergistic effect caused by the high electrical conductivity of N-CDs and the good catalytic activity and high surface area of Cu_2_O.

The effective surface area of N-CDs/GCE, Cu_2_O/GCE and N-CDs/Cu_2_O/Nf/GCE were calculated using different scan rates of cyclic voltammetric responses at 1 mM K_3_[Fe(CN)_6_] in 0.1 M KCl supporting electrolyte. The Randles–Sevcik equation is used as follows: [39]

$$i_{p}=\left(2.69\times 10^{5}\right)A_{eff}D^{\frac{1}{2}}n^{\frac{3}{2}}\upsilon^{\frac{1}{2}}C$$

where i_p_ is the anodic peak current (A), A*_eff_* is an effective surface area (cm^2^), D is diffusion coefficient of potassium ferricyanide (7.60 × 10^−6^ cm^2^ s^−1^) [40], υ is the scan rate (V s^−1^), n is the number of electrons involved in the electrochemical reaction of potassium ferricyanide (n = 1) and C is the bulk concentration of the redox probe (mol. cm^-3^). By taking the slope of the plot of i_pa_ vs ν^1/2^ (Figure 3B) into account, the effective surface area of N-CDs/Cu_2_O/Nf/GCE was found to be 0.1582 cm^2^ which was higher than that of the effective surface area of N-CDs/GCE (00756 cm^2^) and Cu_2_O/GCE (0.0945 cm^2^). This evidence demonstrates that the N-CDs/Cu_2_O/Nf/GCE possesses a good electrochemical effective surface area.

### 3.3. Electrochemical Performance of N-CDs/Cu_2_O/Nf/GCE towards CPZ Oxidation

**Figure 4:**
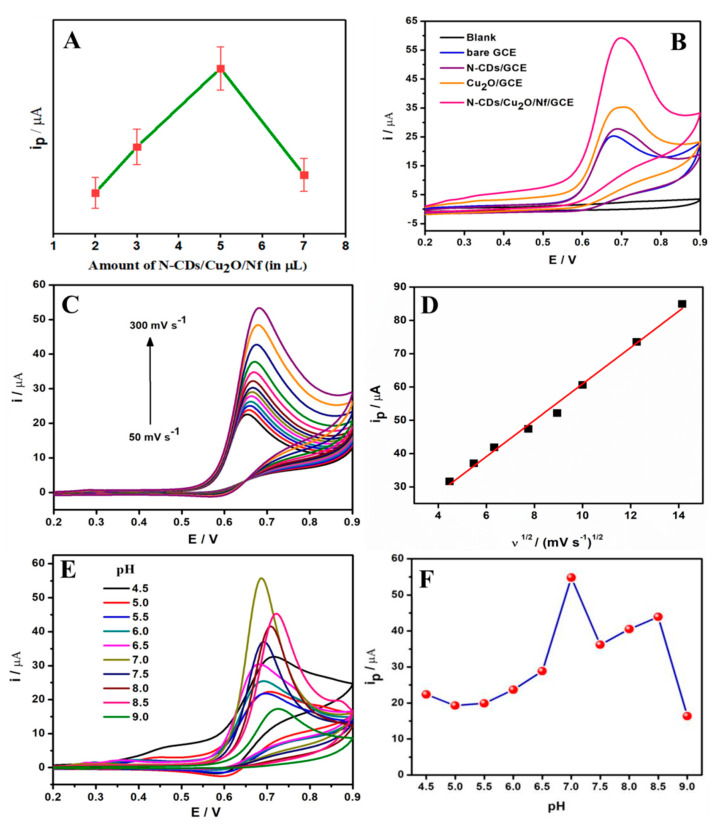
(**A**) The plot of loaded amount of N-CDs/Cu2O/Nf composite onto the surface of GCE vs. i_p_, (**B**) cyclic voltammograms of different modified electrodes at a scan rate of 100 mV s^−1^ and (**C**) various scan rates at N-CDs/Cu2O/Nf/GCE in 0.1 M PBS (pH 7.0) containing 1 mM CPZ, (**D**) calibration plot of square root of scan rate vs. peak current, (**E**) cyclic voltammograms of CPZ at different pH at a scan rate of 100 mV s^−1^ and (**F**) the plot of pH (4.5–9.0) vs. i_p_ of CPZ.

To estimate the influence of the amount of N-CDs/Cu_2_O/Nf on the surface of GCE, the electrochemical performance of N-CDs/Cu_2_O/Nf/GCE in 0.1 M PBS (pH 7.0) containing 1mM CPZ was recorded. Figure 4A displays the electrocatalytic oxidation peak current of CPZ is nonlinear over the increasing amount of N-CDs/Cu_2_O/Nf. Obviously, the highest peak current of CPZ was achieved when 5 µL of N-CDs/Cu_2_O/Nf was applied onto the surface of GCE. Thus, the optimized concentration was chosen for ultrasensitive detection of CPZ.

The electrochemical response of CPZ at modified and unmodified electrodes was verified using CV technique. Figure 4B shows cyclic voltammograms of 1 mM CPZ in 0.1 M PBS (pH 7.0) at GCE, N-CDs/GCE, Cu_2_O/GCE and N-CDs/Cu_2_O/Nf/GCE. Apparently, no characteristic peak was detected in 0.1 M PBS without CPZ in the given window of potential. The bare GCE exhibited an irreversible voltammogram with poor peak current for CPZ, while the N-CDs/GCE displayed higher electrochemical oxidation response due to the better conductive and stable nature of N-CDs [41]. A notable enlargement in the peak current of CPZ was observed at Cu_2_O/GCE when compared with GCE and N-CDs/GCE owing to the good electrons transfer property at the electrode surface that is endowed by the catalytic activity of Cu_2_O. Beyond that, N-CDs/Cu_2_O/Nf/GCE presented the superior peak current among all the electrodes with a shifting towards the less positive potential for electro-oxidation of CPZ, which is attributed to the high surface area of N-CDs/Cu_2_O on the surface of modified electrode.

The influence of scan rate on the oxidation peak current of CPZ was examined and Figure 4C displays the changes of anodic peak current with an increasing scan rate of 1 mM CPZ from 50 to 300 mV s^−1^. The linear relationship between oxidation peak current and the square root of scan rate was evident in Figure 4D. Moreover, with the increasing of scan rate, an obvious shift of the anodic peak potentials towards the positive was emerged, indicating the limitation of charge transfer kinetics. The electrochemical observations revealed that the electro-oxidation of CPZ was a diffusion-controlled irreversible process at the surface of N-CDs/Cu_2_O/Nf/GCE.

The electrochemical reactions of organic compounds are usually carried out with the involvement of protons which have a significant impact on speeding up the reactions. The pH of supporting electrolyte generally influences the electrochemical reaction of CPZ by shifting its oxidation potentials towards positive side due to the acid dissociation of CPZ. Hence, to optimize the pH of supporting electrolyte for the electrochemical oxidation of CPZ at the composite electrode, a cyclic voltammetric investigation was performed in 0.1 M PBS with the pH ranging from 4.5 to 9.0 (Figure 4E). The negligible shift of CPZ peak potentials at various pH conditions indicates that the electro-oxidation of CPZ is a simple electron-transfer process rather than a proton transfer process. However, the anodic peak current of CPZ strongly depends on the pH of the supporting electrolyte. Figure 4F displays the relationship between the pH of analytes and their corresponding anodic peak currents for N-CDs/Cu_2_O/Nf composite electrode, in which the maximum peak current was attained at pH 7.0. Hence, the PBS with a pH of 7.0 was selected as an optimized condition throughout the following electrochemical study of CPZ at N-CDs/Cu_2_O/Nf composite-based sensor.

**Figure 5:**
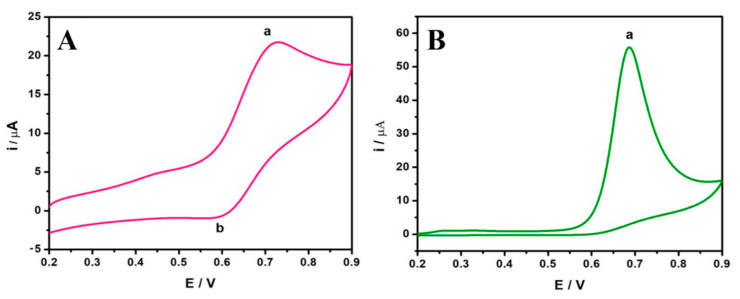
Cyclic voltammograms of 1 mM CPZ in 0.1 M PBS with (**A**) pH 4.5 and (**B**) pH 7.0.

**Scheme 2:**
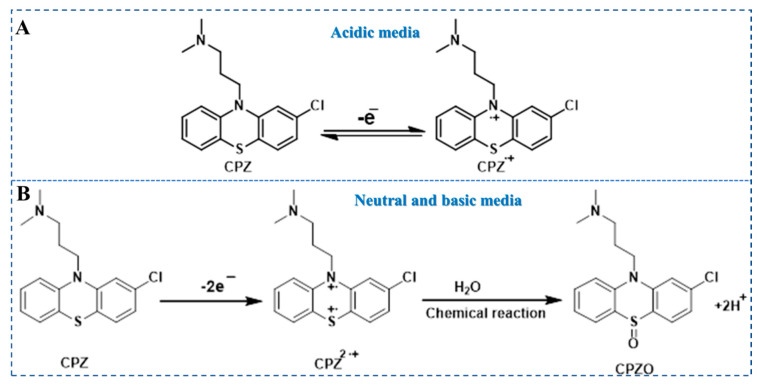
Possible electrochemical mechanism of CPZ in (**A**) acidic media, (**B**) neutral and basic media at N-CDs/Cu_2_O/Nf/GCE.

According to the results in Figure 4E, the redox peaks with reversible cyclic voltammogram can be observed from pH 4.5 to 6.5, whereas only oxidation peaks are seen from pH 7.0 to 9.0. A reversible redox system in acidic media is shown in Figure 5A. An oxidation peak was observed in the forward scan at 0.730 V and the counterpart reduction peak was attained at 0.571 V, which is due to the influence of the pH of supporting electrolyte on the peak potentials of redox peaks. The oxidation peak appeared in the forward scan is attributed to the formation of CPZ cation radical (CPZ**^·^**^+^) through one-electron oxidation of CPZ, whereas the reduction peak in reverse scan relates to the reduction of CPZ**^·^**^+^ by a slow disproportionation reaction (Scheme 2A) [42]. The CPZ cation radical is more stable in acidic media. However, Figure 5B displays an irreversible system in neutral and basic media. Obviously, only oxidation peak at 0.687 V in the forward scan was observed, which signifies that with the increasing of pH values, direct conversion of CPZ to CPZ^2·+^ would take place and make the process unstable. It gets a conversion to the final product by taking oxygen from the water molecule by a chemical reaction without any electrochemical-reduction (Scheme 2B). 

### 3.4. Analytical Performance

**Figure 6:**
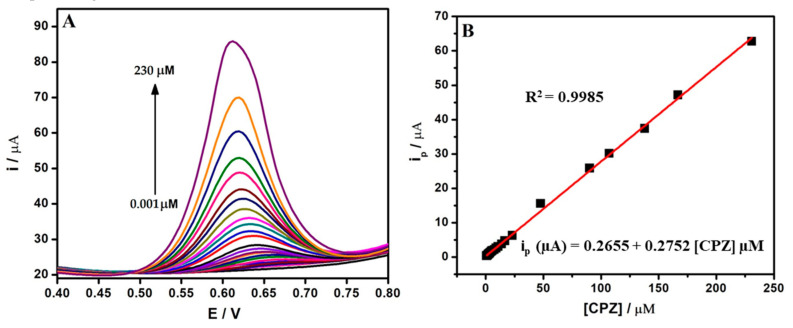
(**A**) Differential pulse voltammograms of CPZ with different concentrations in 0.1 M PBS (pH 7.0), (**B**) calibration plot of concentration of CPZ vs i_p_.

The analytical electrochemical performance of N-CDs/Cu_2_O/Nf/GCE was studied at various concentrations of CPZ under the optimal experimental conditions using differential pulse voltammetry (DPV) technique. The recorded voltammograms were presented in Figure 6A. It simplifies that there was no prominent peak of CPZ potential appeared in the absence of CPZ. The characteristic oxidation peaks of CPZ (≈ 0.65 V) in 0.1 M PBS (pH 7.0) increased with the increasing concentration of CPZ without affecting the oxidation potential in the range of 0.001 µM and 230 µM. The calibration plot of the CPZ concentration and its corresponding peak current was depicted in Figure 6B. The detection limit was found to be 25 nM (S/N = 3) and is comparable in a dynamic linear range (0.001 to 230 µM), signifying the superior performance of the designed sensor. Additionally, the analytical merits of the present sensor are compared with previously reported sensing protocols of CPZ (Table 1) and enlightening the capability of the developed sensor towards the determination of CPZ. The outstanding analytical response of N-CDs/Cu_2_O/Nf modified electrode can be ascribed to the enhanced surface area of the composite onto the electrode which was produced by the incorporating of N-CDs into Cu_2_O.

### 3.5. Interference Study

**Figure 7:**
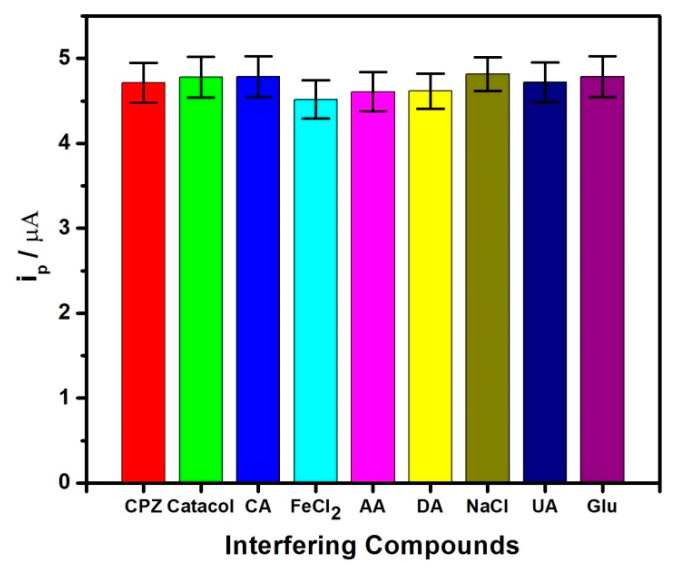
Comparison of the peak current of CPZ in the presence of interfering compounds at N-CDs/Cu_2_O/Nf/GCE (CA: Caffeic Acid; AA: Ascorbic acid; DA: Dopamine; UA: Uric acid; Glu: Glucose).

It is well-known that the anti-interference is one of the most important parameters for electrochemical sensing of CPZ. Some organic compounds and inorganic ions may exist in pharmaceutical and biological samples in the quantification of CPZ. Figure 7 displays the comparison of the peak current of 200 µM CPZ in the presence of 100 folds of common interfering compounds at N-CDs/Cu_2_O/Nf/GCE. The negligible variations of the peak currents for various interfering substances reveal that the developed composite-based sensor has high reliability and good selectivity for ultrasensitive electrochemical sensing of CPZ.

### 3.6. Stability, Repeatability, and Reproducibility

Repeatability refers to the ability of a sensor to produce almost the same signals in consecutive electrochemical measurements. To verify the repeatability of the developed N-CDs/Cu_2_O/Nf composite-based sensor, 30 distinct cyclic voltammetric cycles were recorded with and without CPZ (20 µM) alternately. There was a little difference in anodic peak currents and the peak potentials of CPZ for these repeated tests. The relative standard deviation (RSD) for the measurements was calculated to be 1.53% demonstrating that the sensor has good repeatability. To investigate the reproducibility of the proposed sensor, three N-CDs/Cu_2_O/Nf composite-based sensors were constructed under the same working conditions and RSD of 1.83% was presented for these sensors with 20 µM CPZ, confirming its good reproducibility. The long-term stability of N-CDs/Cu_2_O/Nf composite modified electrode was further determined by storing the electrode for four weeks at 4 °C. Remarkably, the anodic peak current of CPZ, after four weeks, maintained more than 97% of its initial signal, which signifies that the proposed sensor could be used for determination of pharmaceutical samples with acceptable operational stability.

### 3.7. Real Sample Analysis

To further appraise the reliability of the proposed sensor, examination of CPZ was carried out in pharmaceutical formulations (Figure S2) and human urine sample (Figure S3). Before the measurements, 0.1 mL of human urine sample was diluted with 9.0 mL of PBS (pH 7.0) to reduce the matrix effect for obtaining accurate results. The stock solution of tablet sample was prepared with the help of PBS (pH 7.0). Although spiking a known concentration of CPZ, the oxidation peak current of CPZ increased linearly without any peak potential shift. The content of CPZ in both the samples was determined at N-CDs/Cu_2_O/Nf composite modified electrode using the standard addition method. Each addition was measured three times using DPV technique under the optimal working conditions. The obtained results were tabulated in Table 2. The recovery profile reveals that the developed sensor is greatly reliable for determining CPZ in real sample analysis and confirming a promising analytical sensing platform for pharmaceutical drugs.

## 4. Conclusion

In this work, we have developed a versatile strategy for developing a sensing platform for CPZ based on nitrogen-doped carbon dots with cuprous oxide (N-CDs/Cu_2_O) composite. The as-synthesized composite was characterized using physicochemical and electrochemical techniques. With the support of Nafion (Nf), N-CDs/Cu_2_O composite was successfully employed as a sensing material for the detection of CPZ. The composite-based sensor showed excellent performance towards CPZ determination in a dynamic linear range of 0.001 – 230 µM with a detection limit of 25 nM. The developed sensor displayed long-term stability, good anti-interfering property, repeatability, and reproducibility. Additionally, the potential applicability of N-CDs/Cu_2_O/Nf/GCE was successfully verified with satisfactory recoveries in pharmaceutical drug and human urine samples. The successful performance of N-CDs/Cu_2_O/Nf composite sensor was attributed to the synergetic effect of N-CDs and Cu_2_O, which made the effective electron-transfer ability at the surface of electrode. Therefore, the developed sensor can be potentially used for clinical applications.

## Figures and Tables

**Table 1:** Comparison of the analytical merits of N-CDs/Cu_2_O/Nf composite with previously reported sensing materials for electrochemical determination of CPZ.

Sensing Material	Technique	pH	Linear Range (M)	LOD (nM)	Ref.
PNIPAM	DPV	7.0	0.05 µM–7999 µM	16	[43]
β-SnWNRs	Amperometry	7.0	0.01 µM–457 µM	3	[44]
CdO/NPs/IL	Chronoamperometry	7.0	0.1 µM–350 µM	70	[2]
Peas-like SrM	DPV	7.0	0.1 µM–143 µM; 153 µM–1683 µM	28	[45]
BDD	DPV	4.0	0.1 µM–40.0 µM	30	[46]
PTN	DPV	7.0	0.1 µM–130 µM	300	[47]
N-CDs/Cu_2_O/Nf	DPV	7.0	0.001 µM–230 µM	25	Present work

PNIPAM: poly-N-isopropylacrylamide microgel; β-SnWNRs: one-dimensional β-stannous tungstate nanorods; CdO/NPs/IL/CP: CdO/nanoparticles (NPs) ionic liquid carbon paste; SrM: Strontium molybdate; BDD: Boron-doped diamond: PTN: Polythiophene Nanowires.

**Table 2:** Recovery profile of CPZ at N-CDs/Cu_2_O/Nf/GCE in real samples (n = 3).

Sample	S. No	Added (µM)	Found (µM)	Recovery (%)	Bias (%)
Tablet	1	0	Not detected	-	-
	2	10	09.94	99.40	0.6
	3	20	20.22	100.10	1.1
	4	30	30.01	100.03	0.03
Human Urine	1	0	Not detected	-	-
	2	10	09.71	97.10	2.90
	3	20	20.50	102.50	2.50
	4	30	29.31	97.70	2.30

## Supplementary Materials

The following are available online at https://www.mdpi.com/2079-4991/10/8/1513/s1, Figure S1: XPS spectra of N-CDs/Cu_2_O composite, Figure S2: DPV responses for detection of CPZ in pharmaceutical formulations, Figure S3: DPV responses for detection of CPZ in human urine sample.
