# Supplementary material for: A Versatile and Ultrasensitive Electrochemical Sensing Platform for Detection of Chlorpromazine Based on Nitrogen-Doped Carbon Dots/Cuprous Oxide Composite
Source: Nanomaterials (Basel). 2020 Aug 1;10(8):1513. doi: 10.3390/nano10081513 (PMC7466556; doi:10.3390/nano10081513)
Supplement: Supplementary file 1 [file nanomaterials-10-01513-s001.pdf]

A Versatile and Ultrasensitive Electrochemical Sensing Platform for Detection of Chlorpromazine Based on Nitrogen-doped Carbon Dots/Cuprous Oxide Composite

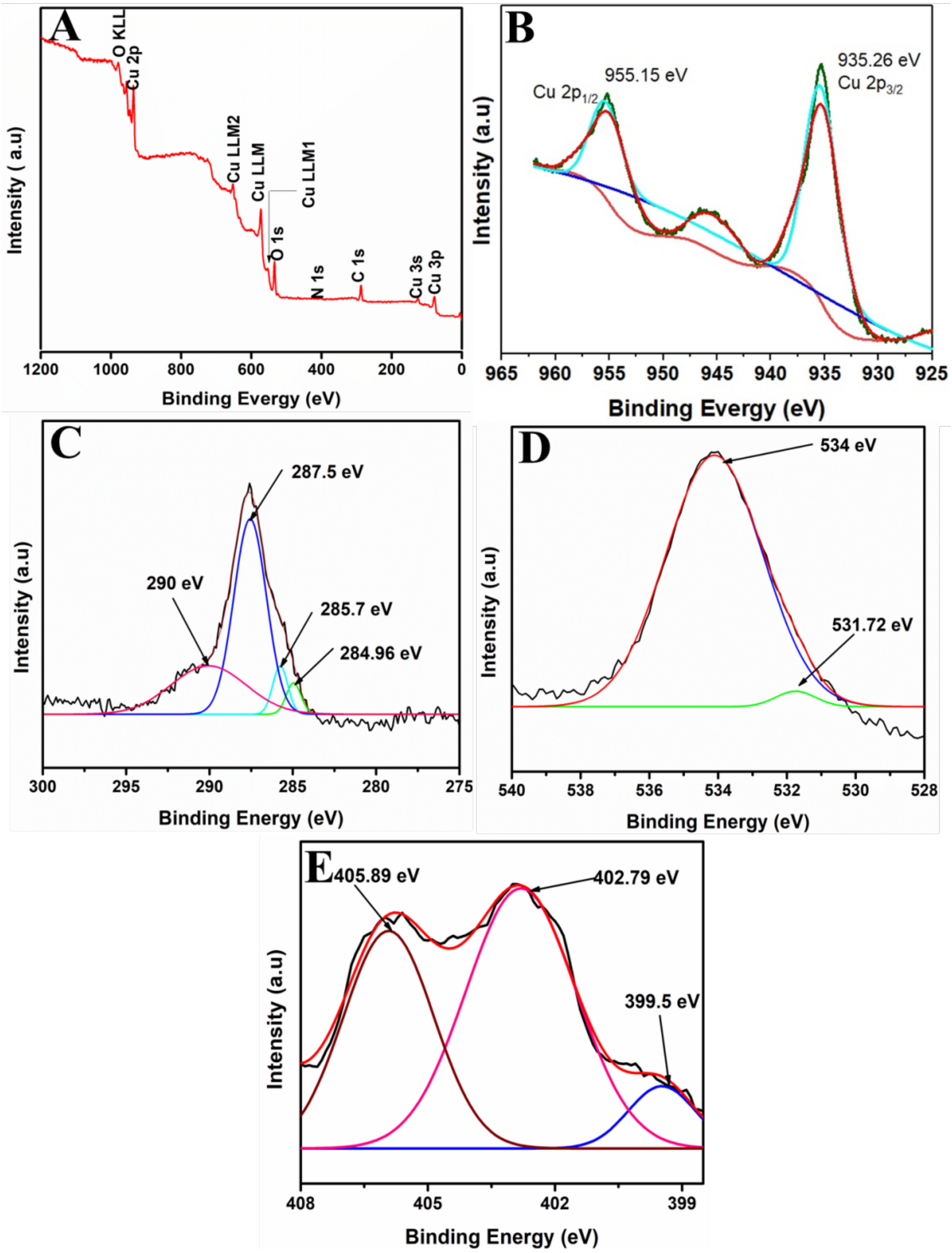

Figure S1. (**A**) XPS survey spectrum of N-CDs/Cu_2O composite, XPS signals of (**B**) Cu 2p, (**C**) C 1s, (**D**) O 1s and (**E**) N 1s.

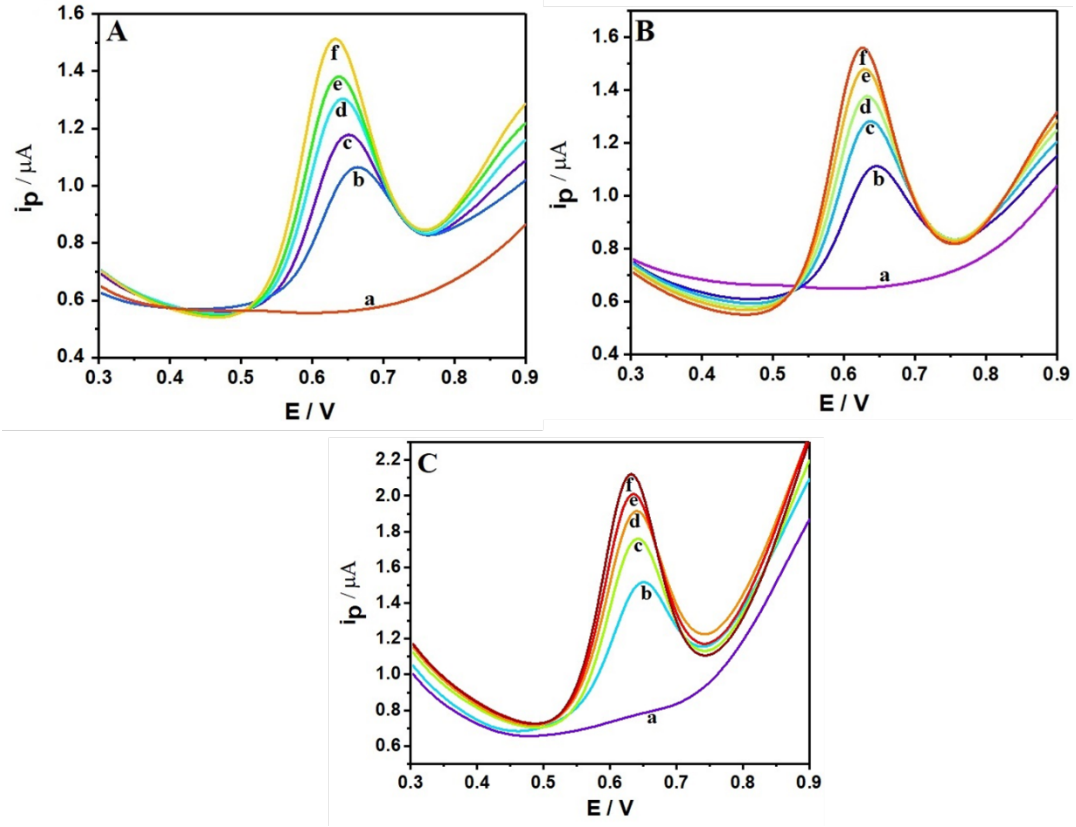

Figure S2. (**A**) Differential pulse voltammetric responses of N-CDs/Cu_2O/Nf/GCE for standard addition of (c) 5 μM, (d) 10 μM, (e) 15 μM and (f) 20 μM of CPZ standard sample to (b) 10 μM tablet sample in 0.1 M PBS (pH 7.0) and (a) blank response; (**B**) Differential pulse voltammetric responses of N-CDs/Cu_2O/Nf/GCE for standard addition of (c) 5 μM, (d) 10 μM, (e) 15 μM and (f) 20 μM of CPZ standard sample to (b) 20 μM tablet sample in 0.1 M PBS (pH 7.0) and (a) blank response; (**C**) Differential pulse voltammetric responses of N-CDs/Cu_2O/Nf/GCE for standard addition of (c) 5 μM, (d) 10 μM, (e) 15 μM and (f) 20 μM of CPZ standard sample to (b) 30 μM tablet sample in 0.1 M PBS (pH 7.0) and (a) blank response.

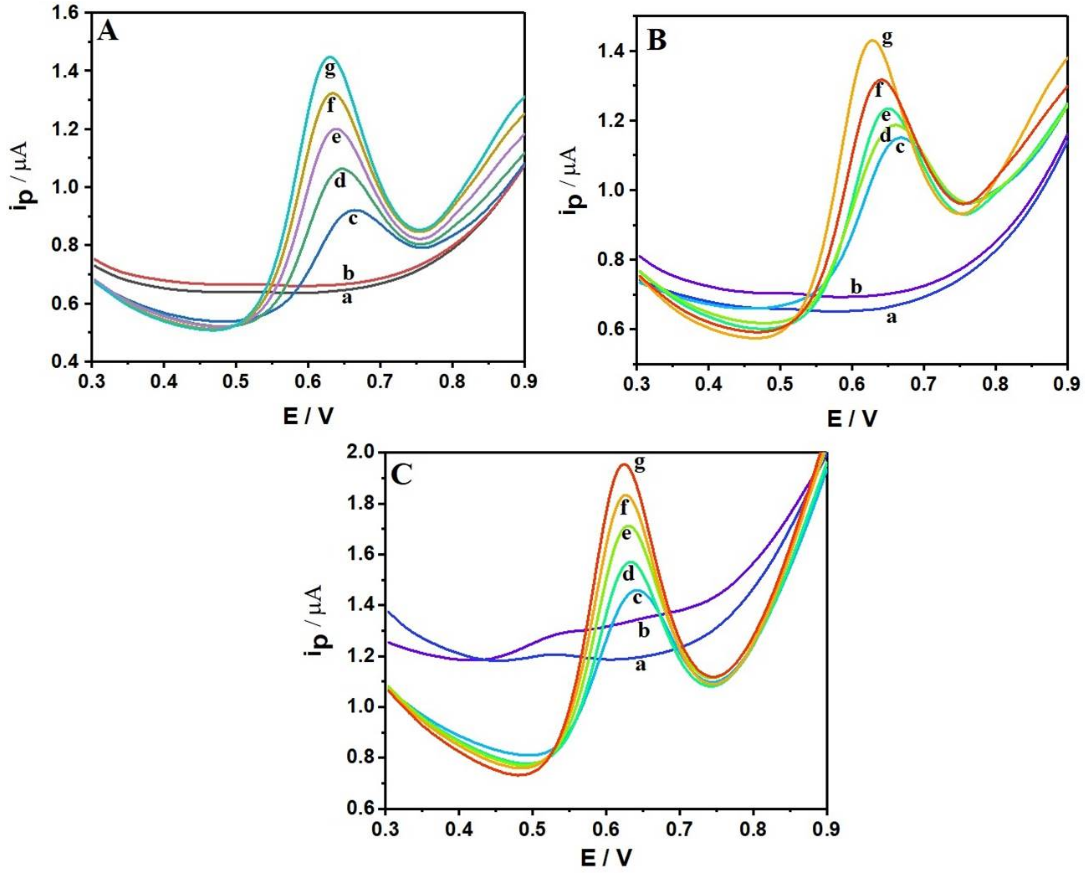

Figure S3. (**A**) Differential pulse voltammetric responses of N-CDs/Cu_2O/Nf/GCE for standard addition of (d) 5 µM, (e) 10 µM, (f) 15 µM and (g) 20 µM CPZ standard sample to (c) 10 µM spiked CPZ sample in 0.1 M PBS (pH 7.0) containing human urine sample and (a) blank response, (b) urine sample response; (**B**) Differential pulse voltammetric responses of N-CDs/Cu_2O/Nf/GCE for standard addition of (d) 5 µM, (e) 10 µM, (f) 15 µM and (g) 20 µM CPZ standard sample to (c) 20 µM spiked CPZ sample in 0.1 M PBS (pH 7.0) containing human urine sample and (a) blank response, (b) urine sample response; (**C**) Differential pulse voltammetric responses of N-CDs/Cu_2O/Nf/GCE for standard addition of (d) 5 µM, (e) 10 µM, (f) 15 µM and (g) 20 µM CPZ standard sample to (c) 30 µM spiked CPZ sample in 0.1 M PBS (pH 7.0) containing human urine sample and (a) blank response, (b) urine sample response.
